# ﻿Caribbean Amphipoda (Crustacea) of Panama. Part I: parvorder Oedicerotidira

**DOI:** 10.3897/zookeys.1159.102034

**Published:** 2023-04-24

**Authors:** Elizabeth L. Durham, Kristine N. White

**Affiliations:** 1 Georgia College & State University, Department of Biological and Environmental Sciences, Milledgeville, GA 31061, USA Georgia College & State University Milledgeville United States of America

**Keywords:** Bocas del Toro, Caribbean, *Hartmanodesnyei*, identification key, new species, Oedicerotidae, Panama, *Synchelidiumpurpurivitellum* sp. nov.

## Abstract

Amphipods in the parvorder Oedicerotidira are burrowers, furrowers, or surface skimmers. Members of the parvorder share a well-developed posteroventral lobe on coxa 4, an equilobate coxa 5, an immensely elongate pereopod 7 that differs in structure from pereopod 6, and an entire telson. Within the parvorder, only the family Oedicerotidae has been documented from Bocas del Toro, Panama, represented by two species. This research documents a range extension for *Hartmanodesnyei* (Shoemaker, 1933) and describes a new species of *Synchelidium* Sars, 1892. An identification key to the species of Caribbean Oedicerotidae of Panama is provided.

## ﻿Introduction

Parvorder Oedicerotidira Lilljeborg, 1865 (Lilljeborg 1865b) is comprised of 302 species, with representative species documented around the world. Members of Oedicerotidira share a well-developed posteroventral lobe on coxa 4, an equilobate coxa 5, an immensely elongate pereopod 7 that differs in structure from pereopod 6, and an entire telson (Lowry and Myers 2017). The parvorder contains three families of burrowing, furrowing, or surface skimming amphipods: Exoedicerotidae Barnard & Drummond, 1982 (20 spp.), Oedicerotidae Lilljeborg, 1865 (262 spp.), and Paracalliopiidae Barnard & Karaman, 1982 (20 spp.). Only one of these families, the Oedicerotidae, is known to occur in the Caribbean Sea and, to date, only six species within that family have been reported from the Caribbean: Aceroides (Patoides) synparis (Barnard, 1964); *Americhelidiumamericanum* (Bousfield, 1973); *Hartmanodesnyei* (Shoemaker, 1933); *Kroyeracarinata* Bate, 1857 (as Monoculodescf.carinatus); *Perioculodescerasinus* Thomas & Barnard, 1985; *Westwoodillalongimana* Shoemaker, 1934 (LeCroy et. al. 2009; Martín et. al. 2013). Of these Caribbean species, only *A.synparis* has been documented from Panamanian waters, occurring at a depth of 850 m (Barnard 1964).

Defining characteristics of amphipods in the family Oedicerotidae include having a well-developed antenna 2, reaching at least half the length of antenna 1; a strong down-curved rostrum; well-developed dorsolateral eyes; coxae 1–3 well-developed, each longer than the previous coxa; a subchelate gnathopod 1; article 3 of gnathopod 2 less than 2 × as long as wide; and a distally attenuate pereopod 7 that is longer and more slender than pereopod 6. Amphipods in the family Oedicerotidae differ from those in the families Exoedicerotidae and Paracalliopidae in having separate urosomite segments and lacking the oblique setal row on the maxilla 2 inner plate (Lowry and Myers 2017). Most species of the parvorder burrow into sediment, but little else is known about their ecology.

Two species of oedicerotid amphipods were collected from Bocas del Toro, Panama, one of which is new to science. Both species are diagnosed and the new species is described herein, and an identification key is provided to distinguish between the three species known from the Caribbean waters of Panama.

## ﻿Materials and methods

Amphipods were collected by hand using a Ziploc bag to scoop up fine sand from Crawl Cay, Bocas del Toro, Panama at depths of 1.5–5.0 m. The sand was elutriated with freshwater to remove amphipods. Live specimens were sorted to morphospecies, placed in clove oil for imaging, and preserved in 99.5% EtOH for later examination. Preserved specimens were transferred to glycerol, measured from the tip of the rostrum to the base of the telson, and dissected under a stereomicroscope. Specimens were illustrated using a Meiji MT5900L phase contrast microscope with an Olympus U-DA drawing tube attached. Illustrations were digitally inked following Coleman (2003) in Adobe Illustrator 2020 using a Wacom Intuos Pro Pen Tablet. Specimens are deposited in the Smithsonian Institution, U.S. National Museum of Natural History (**USNM**).

## ﻿Results

### ﻿Descriptions

**Parvorder Oedicerotidira Lilljeborg, 1865 (Lilljeborg, 1865b)**

**Superfamily Oedicerotoidea Lilljeborg, 1865 (Lilljeborg, 1865a)**

#### ﻿Family Oedicerotidae Lilljeborg, 1865 (Lilljeborg, 1865b)

##### ﻿Genus Hartmanodes

Taxon classificationAnimaliaAmphipodaOedicerotidae

Bousfield & Chevrier, 1996

147FB667-622E-54E6-A969-D84D5031D36D

###### Generic diagnosis.

Antenna 1 shorter than antenna 2; male antenna 2 much longer than that of female; head, rostrum large, apex deflexed. Gnathopods 1 and 2 not sexually dimorphic; gnathopod 1 carpus broad, propodus long, ovate; Gnathopod 2 subchelate, carpus narrow, propodus elongate, narrowing distally. Pereopod 5 coxa large, deep, equilobate. Pereopod 7 basis with small posterodistal lobe. Telson short, apex truncate or emarginate.

##### ﻿ Hartmanodes nyei

Taxon classificationAnimaliaAmphipodaOedicerotidae

(Shoemaker, 1933)

F037AE9B-DF2C-5C05-8C57-5C33078DAEC7

Figs 1 , 2 , 6A

Monoculodes nyei Shoemaker, 1933: 9, fig. 5.
Hartmanodes nyei : Bousfield and Chevrier 1996: 92; LeCroy 2000: 169, fig. 205.

###### Material examined.

Panama • 1 ♀, 4.0 mm; Bocas del Toro, Crawl Cay; 9.2475°N, 82.1290°W; depth 5 m, in sand; 12 Aug 2021; K.N. White leg; USNM 1522425 • 1 ♂, 3.5 mm; same station data as for preceding; USNM 1522426 • 4 ♀; same station data as for preceding; USNM 1522427 • 1 ♀, 1 ♂, 2 juvenile; Bocas del Toro, Crawl Caye; 9.2376°N, 82.1438°W; depth 2 m, in sand, 11 Aug 2021; K.N. White leg.; USNM 1522428.

###### Diagnosis.

Antenna 1 of female subequal to peduncle of antenna 2; antenna 2 of male much longer than that of female; head, anterodorsal angle broadly subquadrate. Pereopods 3 and 4 propodus subrectangular, dactylus elongate, slender; pereopods 5 and 6 dactylus elongate, subequal to propodus in length; pereopod 7 basis posterior margin with several short setae, carpus and propodus, posterior margin with several spine groups, dactylus elongate, slender.

**Figure 1.**
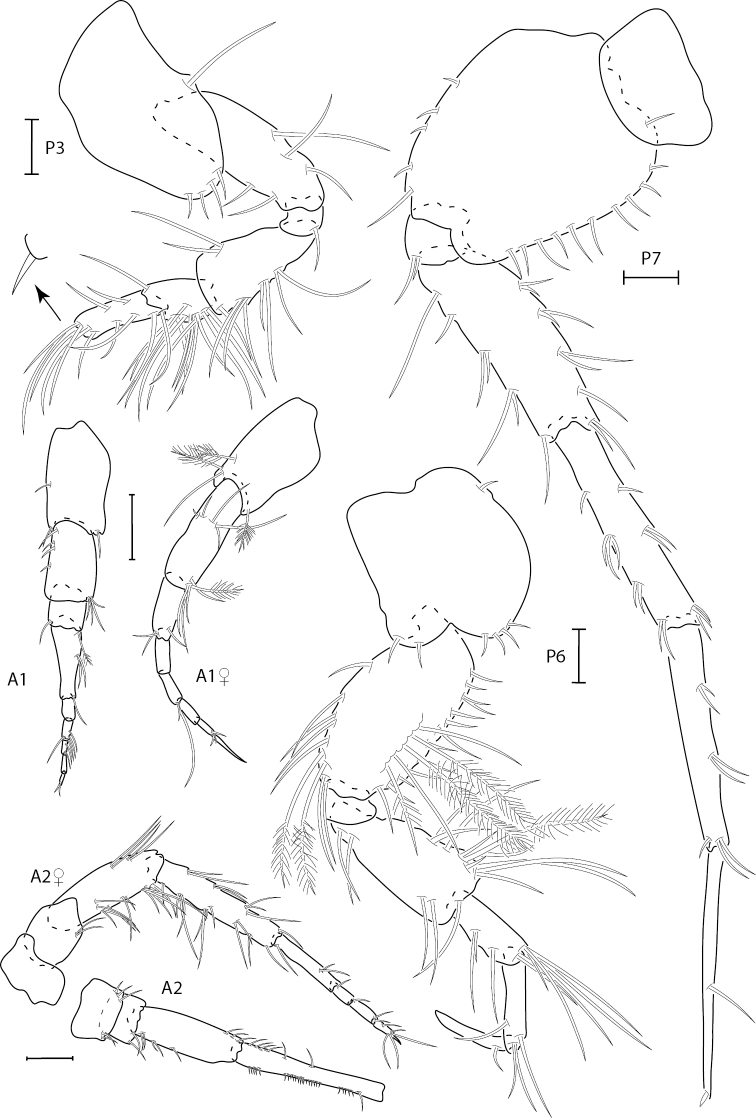
*Hartmanodesnyei*, female, 4.0 mm, pereopod 3, pereopod 3 dactyl with setae removed, pereopod 6 and 7, antennae 1 and 2; male, 3.5 mm, antenna 1 and antenna 2 (broken). Scale bars: 0.1 mm.

###### Distribution.

U.S.A.: Gulf of Mexico (Rakocinski et al. 1993), South Florida (Shoemaker 1933; Thomas 1993), Pacific California (Barnard 1962; Bousfield and Chevrier 1996); South America: Brazil (Shoemaker 1933); Central America: Belize (Thomas 1993), Panama (present study).

**Figure 2.**
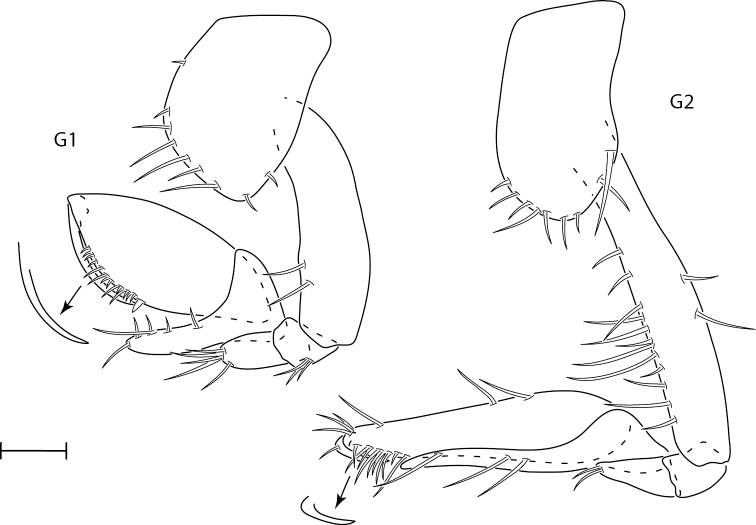
*Hartmanodesnyei*, female, 4.0 mm, gnathopods 1 and 2 lateral. Scale bar 0.1 mm.

###### Ecology.

These amphipods burrow into sand in shallow subtidal habitats.

##### ﻿Genus Synchelidium

Taxon classificationAnimaliaAmphipodaOedicerotidae

Sars, 1892

1FE4CE85-2570-5412-B6CF-5ABAAA414F14

###### Generic diagnosis.

Antenna 1 shorter than antenna 2; male antenna 2 much longer than that of female; head, rostrum strong, distally deflexed. Gnathopods 1 and 2 not sexually dimorphic; gnathopod 1 carpus elongate, slender, propodus broad; Gnathopod 2 chelate, carpal lobe slender, propodus elongate. Pereopod 5 coxa medium, deep, equilobate. Pereopod 7 basis lacking or with weak posterior lobe. Telson short, apex emarginate or rounded.

##### ﻿ Synchelidium purpurivitellum sp. nov.

Taxon classificationAnimaliaAmphipodaOedicerotidae

BCECAA45-5BE1-57E7-B9A2-83D8562C9053

https://zoobank.org/117F5F75-7DE0-403D-8C9E-34DCB11C8EC1

Figs 3 , 4 , 5 , 6B

###### Type locality.

Bocas del Toro, Panama: Crawl Cay, 9.2475°N, 82.1290°W, depth 5 m, in sand.

###### Material examined.

***Holotype***: Panama • 1 ♀, 2.3 mm; Bocas del Toro, Crawl Cay; 9.2475°N, 82.1290°W; depth 5 m, in sand; 12 Aug 2021; K.N. White leg; USNM 1522429.

***Paratypes***: Panama • 1 ♂, 2.0 mm; same station data as for preceding; USNM 1522430 • 1 ♀, 2.0 mm; same station data as for preceding; USNM 1522431.

***Other material***: Panama • 2 ♀, 1 ♂, 3 juvenile, same station data as for preceding; USNM 1522432.

###### Diagnosis.

Gnathopod 1 propodus, palm regularly toothed. Gnathopod 2 propodus slender, 6 × length of dactyl. Pereopod 3 propodus with anteroproximal margin longer than anterodistal margin, dactylus short, stubby. Coxa 4 posteroventral angle slightly produced. Coxa 6 posteroventral angle narrowly rounded. Pereopod 7 merus with spines on posterior margin slightly shorter than width of article. Epimeron 3 anteroventral margin narrowly produced. Telson thickened dorsoventrally, narrowing distally, apex subtruncate with two medium setae dorsolaterally, two short setae medially.

**Figure 3.**
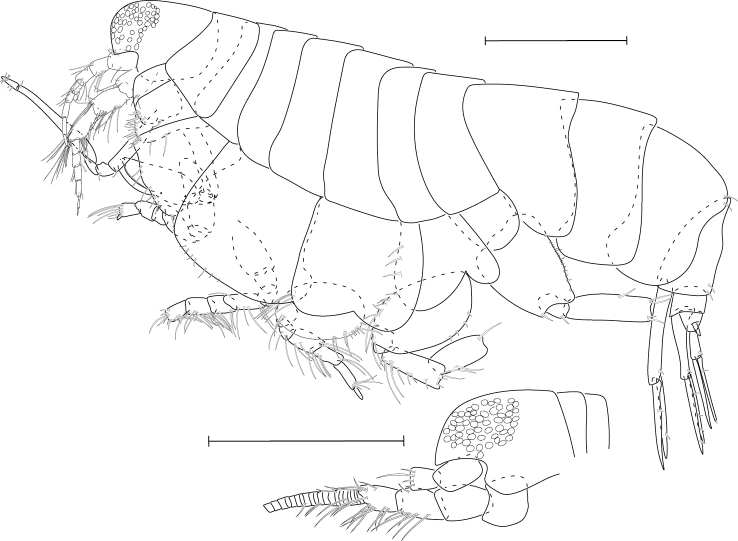
*Synchelidiumpurpurivitellum* sp. nov. holotype female, 2.3 mm, habitus; paratype male, 2.0 mm: head, antennae broken. Scale bars: 0.5 mm.

###### Description.

**Female** (holotype, 2.3 mm). ***Head*.** Rostrum deflexed, reaching ventral margin of head, not surpassing article 1 of antenna 1. Eyes large, covering entire anterior portion of head. Antenna 1 length surpassing peduncle of antenna 2, moderately setose, peduncle segments subequal; flagellum 5-articulate. Antenna 2 is 1.2 × length of antenna 1, flagellum 5-articulate. Maxilliped, inner plate with four apical setae, outer plate with four or five distomedial, marginal setae. Lower lip, inner lobes rounded, outer lobes with large gape, apically setose. Maxilla 1 outer lobe with five apical plumose setae; palp bi-articulate, with three apical setae. Maxilla 2 inner plate with two apical setae, outer plate with three apical setae. Mandibles similar, incisors dentate; left mandible lacinia mobilis 6-dentate, right mandible lacinia mobilis 4-dentate; four accessory spines; molar process small; palp tri-articulate, article 2 with three setae, article 3 subequal in length with article 1, with two or three setae. Upper lip asymmetrical, apically setose.

**Figure 4.**
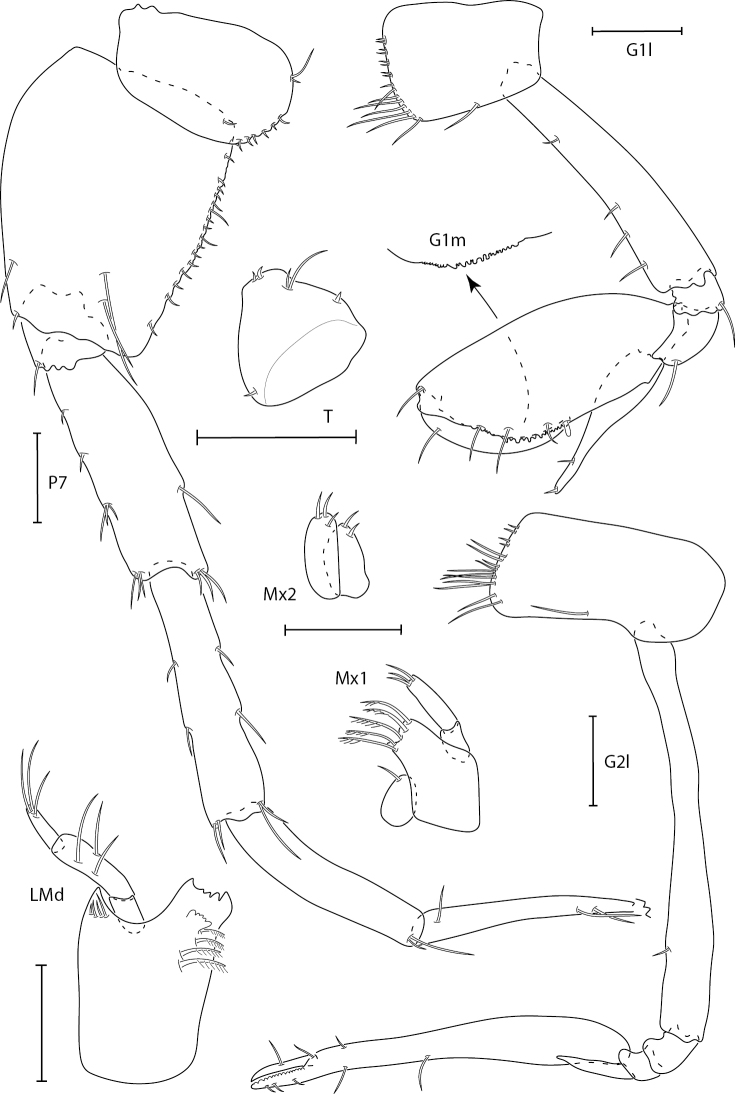
*Synchelidiumpurpurivitellum* sp. nov. holotype female, 2.3 mm, gnathopods 1 and 2 lateral, G1 medial palm, left mandible, maxilla 1 and 2; paratype male, 2.0 mm: pereopod 7 (dactyl broken), telson. Scale bars: 0.1 mm.

***Pereon*.** Coxae weakly setose on distal margin; coxae 1–3 subrectangular; coxa 4 subquadrate, slightly produced posterodistally. Gnathopod 1 subchelate; basis with few short setae on anterior margin; merus not expanded; carpal lobe reaching palmar angle, with two distal setae; propodus ovate, palm oblique, regularly toothed, defined by blunt tipped spine; dactylus reaching palmar angle. Gnathopod 2 chelate; basis slender, with one short seta on anterodistal margin; propodus minutely toothed on cutting edge of fixed finger, sparsely setose; total length of dactylus one-sixth of propodus, smooth. Pereopods 3 and 4 bases slender, with long plumose setae on anterodistal and posterodistal margins; propodus with anteroproximal margin longer than anterodistal margin; dactylus short, stout, one-fourth length of propodus. Pereopod 5 basis anterior margin with distally located plumose setae and a single row of medial plumose setae along the midline; propodus subequal to carpus. Pereopod 6 moderately setose; propodus subequal to carpus. Pereopod 7 basis subrectangular, lacking posterodistal lobe, posterior margin lined with short setae; dactylus styliform, at least as long as propodus (broken).

**Figure 5.**
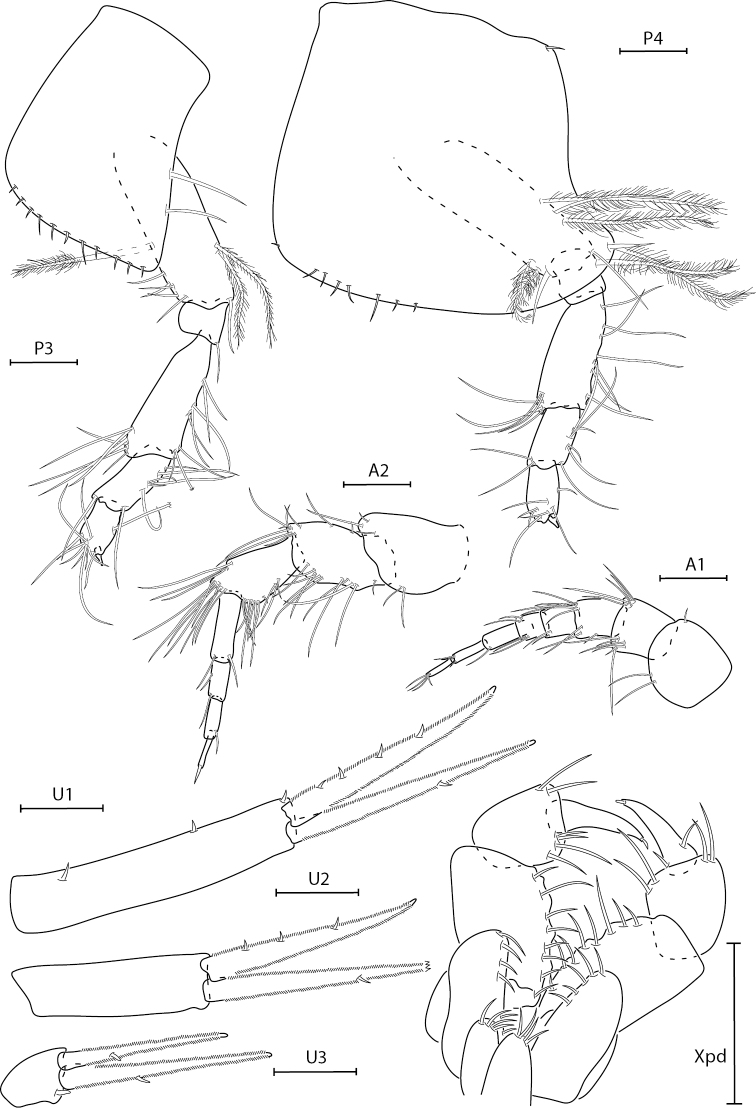
*Synchelidiumpurpurivitellum* sp. nov. holotype female, 2.3 mm, antennae 1 and 2, pereopods 3 and 4, uropods 1–3; paratype male, 2.0 mm: maxilliped. Scale bars: 0.1 mm.

***Pleon*.** Epimera 1–3 margins smooth, bare; epimeron 3, posteroventral margin evenly rounded. Uropod 1, peduncle 1.2 × length of outer ramus, inner and outer rami subequal in length, inner ramus with one robust marginal seta, outer ramus with four robust setae, inner and outer rami lined with fine setae. Uropod 2 peduncle subequal in length to outer ramus, inner ramus broken, inner ramus with one robust marginal seta, outer ramus with three robust setae, inner and outer rami lined with fine setae. Uropod 3 peduncle 0.3 × length of outer ramus, inner ramus 1.2 × length of outer ramus, each ramus with one robust seta, lined with fine setae. Telson thickened dorsoventrally, narrowing distally, apex subtruncate with two medium setae dorsolaterally, two short setae medially.

**Figure 6.**
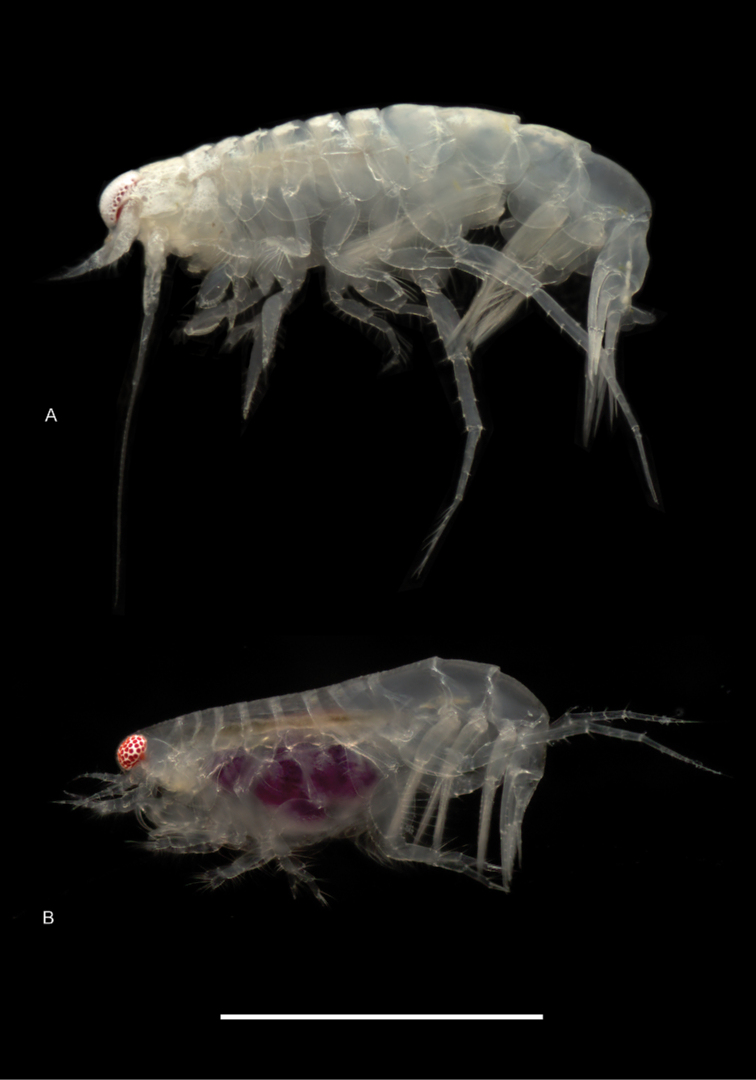
Photographs of live specimens **A***Hartmanodesnyei*, male **B***Synchelidiumpurpurivitellum* sp. nov. female. Scale bar: 1.0 mm.

**Male** (paratype, 2.0 mm). Similar in all aspects to the female with the exception of the following: Eye slightly larger; antenna 2 flagellum elongate, at least 0.5 × body length (broken); gnathopods 1 and 2 bases slightly wider than in female.

###### Etymology.

After the Latin *purpur*, meaning purple and *vitellum*, meaning yolk and referring to the striking purple color of the eggs in the brood pouch of females of this species.

###### Distribution.

Panama: Bocas del Toro (present study).

###### Ecology.

These amphipods burrow into sand in shallow subtidal habitats.

###### Remarks.

*Synchelidiumpurpurivitellum* sp. nov. is similar to the geographically close species *Americhelidiumamericanum* in many aspects, but differs in having a strongly toothed gnathopod 1 propodal palm (smooth in *A.americanum*), a broadly rounded posteroventral corner of epimeron 2 (produced in *A.americanum*), and lacking the posterodistal lobe found on the basis of pereopod 7 in *A.americanum*.

Within the genus, *Synchelidiumpurpurivitellum* sp. nov. is most similar to *Synchelidiummaculatum* Stebbing, 1906, sharing the long gnathopod 1 carpus and toothed propodus palm, minute pereopod 3 and 4 dactyls, and rounded posteroventral corner of epimeron 2. It differs from *S.maculatum* in having shorter antenna 1 flagellum, subrectangular gnathopod 1 propodus (ovate in *S.maculatum*), and subtruncate telson (rounded in *S.maculatum*). The subtruncate telson distinguishes *S.purpurivitellum* sp. nov. from all other *Synchelidium* species, but this character most resembles *Synchelidiumintermedium* Sars, 1892, which has a truncate telson.

### ﻿Identification Key to the Caribbean Oedicerotae of Panama

**Table:** 

1	Gnathopod 1, carpal lobe slender; gnathopod 2 chelate; pereopods 3 and 4, dactylus short	***Synchelidiumpurpurivitellum* sp. nov**
–	Gnathopod 1, carpal lobe broad; gnathopod 2 subchelate; pereopods 3 and 4, dactylus long	**2**
2	Eye well-developed; pereopods 3 and 4, carpus subquadrate, not produced, subequal in width to propodus; pereopod 7, basis without facial setae; telson apically truncate	***Hartmanodesnyei***
–	Eye absent; pereopods 3 and 4, carpus posteroventrally produced, 3 × as wide as propodus; pereopod 7, basis with facial setae; telson apically convex	**Aceroides (Patoides) synparis**

## ﻿Discussion

The results of this study increase the number of Caribbean oedicerotid amphipods known from Panama to three, with the documentation of a new species and a range extension to include Panama for *H.nyei*. *Hartmanodesnyei* has previously been reported from the western Atlantic Ocean, including the Caribbean Sea (Shoemaker 1933; Rakocinski et al. 1993; Thomas 1993; LeCroy 2000), with two questionable records in the Pacific Gulf of California (Barnard 1962; Bousfield and Chevrier 1996).

The genus *Synchelidium* now contains eight species worldwide, with *Synchelidiumpurpurivitellum* sp. nov. being the first Caribbean species of the genus. *Americhelidiumamericanum* was reported from the Caribbean as *Synchelidiumamericanum* before the closely related genus *Americhelidium* Bousfield & Chevrier, 1996 was erected (Thomas 1993). Bousfield and Chevrier (1996) designated *Americhelidium* as a North Pacific coastal marine genus, including *A.americanum*, and *Synchelidium* as a European Atlantic genus. Despite this designation, *Synchelidiumpurpurivitellum* sp. nov. is placed in the genus based on the strongly toothed gnathopod 1 propodus, broadly rounded epimeron 2, and the lack of a posterodistal lobe on the basis of pereopod 7, all of which are the diagnostic characters selected by Bousfield and Chevrier (1996) to distinguish this genus from *Americhelidium*. It is likely that the genera have a wider distribution, but have yet to be documented properly, given the similarities among the species, which would explain its presence in Caribbean Panama. Future investigation of additional material, including type material will aid in understanding the relationships between species of the *Americhelidium* and *Synchelidium*. Documenting these sand burrowing species from Bocas del Toro may allow their inclusion in future applied studies, including studies on trophic interactions, habitat-use, and population or community analyses.

## ﻿Funding

Funding for this study was provided by a National Science Foundation grant: Collaborative Research: ARTS: Understanding Tropical Invertebrate Diversity Through Integrative Revisionary Systematics and Training (1856421). Publication costs were provided by the Georgia College & State University GC Journeys Program.

## Supplementary Material

XML Treatment for Hartmanodes

XML Treatment for Hartmanodes nyei

XML Treatment for Synchelidium

XML Treatment for Synchelidium purpurivitellum

## Supplementary materials

Supplementary material 1 Collection data for Caribbean Oedicerotidira of Panama Data type COL (.xlsx file) https://binary.pensoft.net/file/841941This dataset is made available under the Open Database License (http://opendatacommons.org/licenses/odbl/1.0/). The Open Database License (ODbL) is a license agreement intended to allow users to freely share, modify, and use this Dataset while maintaining this same freedom for others, provided that the original source and author(s) are credited.Elizabeth L. Durham, Kristine N. White
